# Supplementary material for: Mining alternative splicing patterns in scRNA-seq data using scASfind
Source: Genome Biol. 2024 Jul 29;25:197. doi: 10.1186/s13059-024-03323-6 (PMC11285346; doi:10.1186/s13059-024-03323-6)
Supplement: Supplementary file 1 — Additional file 1: Supplementary figures S1–S12 for the publication “Mining alternative splicing patterns in scRNA-seq data using scASfind” [file 13059_2024_3323_MOESM1_ESM.pdf]

Mining alternative splicing patterns in scRNA-seq data using scASfind

Yuyao Song[1,2], Guillermo Parada[1,3], Jimmy Tsz Hang Lee[1], Martin Hemberg[1,4*]

[1]Wellcome Sanger Institute, Hinxton CB10 1SA, UK

[2]European Molecular Biology Laboratory-European Bioinformatics Institute, Hinxton CB10 1SD, UK

[3]Donnelly Centre, University of Toronto, Toronto, ON M5S 3E1, Canada

[4]The Gene Lay Institute of Immunology and Inflammation, Brigham and Women's Hospital, Massachusetts General Hospital, and Harvard Medical School, Boston MA 02115, USA

[*]Corresponding author: mhemberg@bwh.harvard.edu

Supplementary figure S1-S12

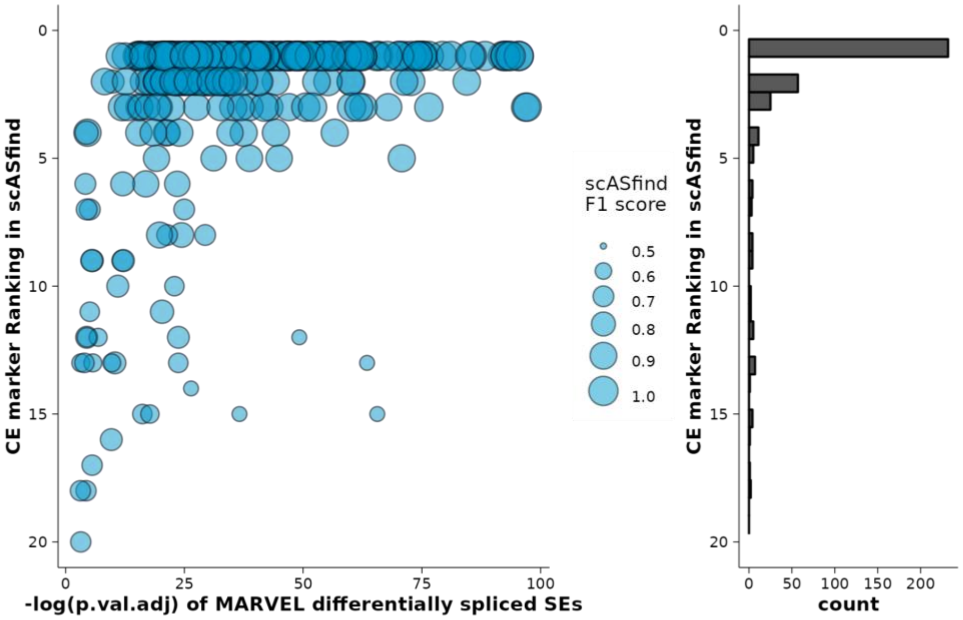

Fig S1 Comparison of MARVEL (1) **differentially spliced SEs with scASfind** (2) **CE markers.** Each dot represents a core exon detected by both methods as differentially spliced between a pair of cell types in the mouse cortex data (3). The dot size corresponds to the F1 score in scASfind, and the count of dots per scASfind rank is shown in the bar chart. CE, core exon; SE, skipped exon.

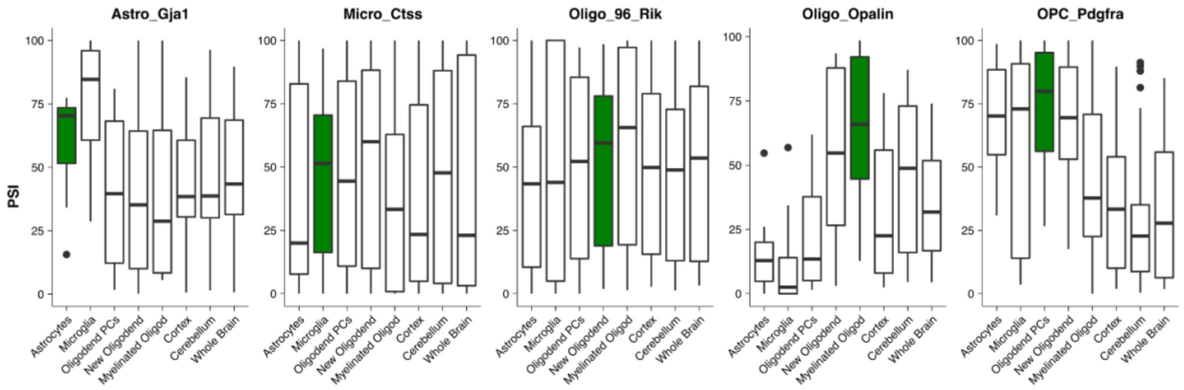

Fig S2 Evaluation of marker nodes from five cell types in the mouse cortex data (3) **using VastDB** (4)**.** For each tissue in VastDB we calculated the PSI for the top 10 cell type marker nodes reported by scASfind. The box encompasses the 25th-75th percentile, and the whiskers extend to the 5th and 95th percentiles with outliers shown as dots. The box which should provide the best match based on the annotated labels is highlighted in green. PSI, percent spliced-in.

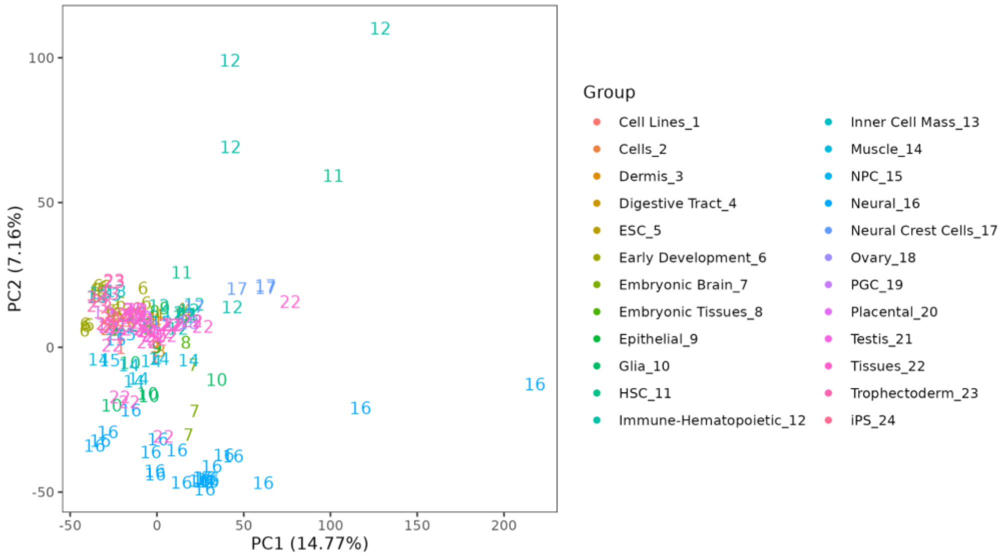

Fig S3 scASfind markers in VastDB show neural specificity. We calculated the top 500 marker splicing nodes for each cell type from scASfind analysis of the mouse cortex data. The VastDB PSI values of these marker nodes for various tissues were then subtracted and subjected to probabilistic principal component analysis. The first two principal components are represented, showing that neuronal tissues and immune cells are distinct from other tissue types. PC, principal component.

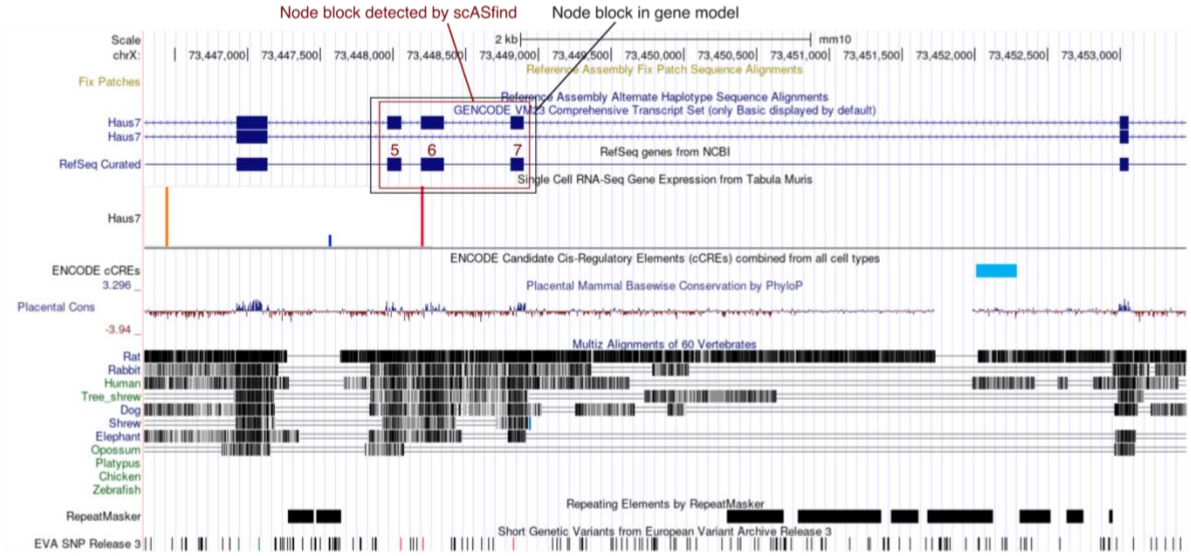

Fig S4: Reference assembly of *Haus7* in the mouse genome from the UCSC genome browser (5)**.** Showing the two alternative isoforms with node blocks. Node block detected by scASfind is indicated in a dark red box while the node block in the gene annotation is shown with a black box. Numbers are node numbers in scASfind.

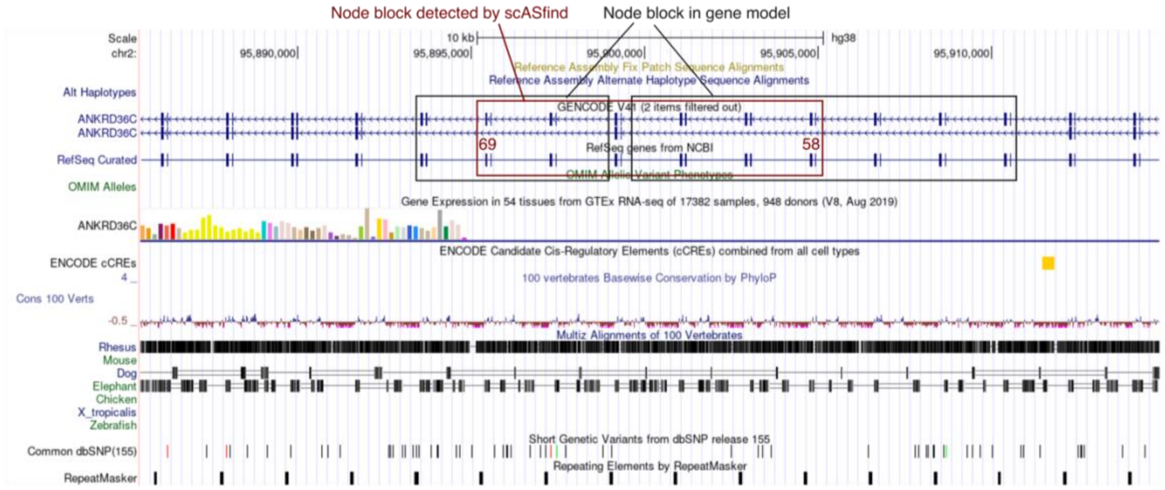

Fig S5: Reference assembly of *ANKRD36C* in the human genome from the UCSC genome browser. Showing the two alternative isoforms with node blocks. Node block detected by scASfind is indicated in a dark red box while the node blocks in the gene annotation are shown with a black box. Numbers are node numbers in scASfind.

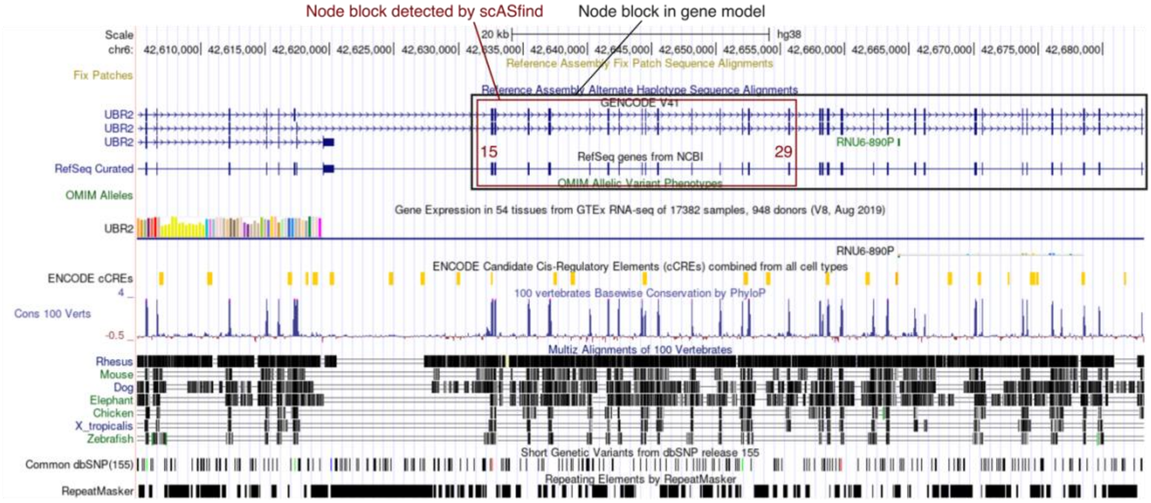

Fig S6: Reference assembly of *UBR2* in the human genome from the UCSC genome browser. Showing the three alternative isoforms of which one results in early termination, in line with scASfind results. Node block detected by scASfind is indicated in a dark red box while the node block in the gene annotation is shown with a black box.

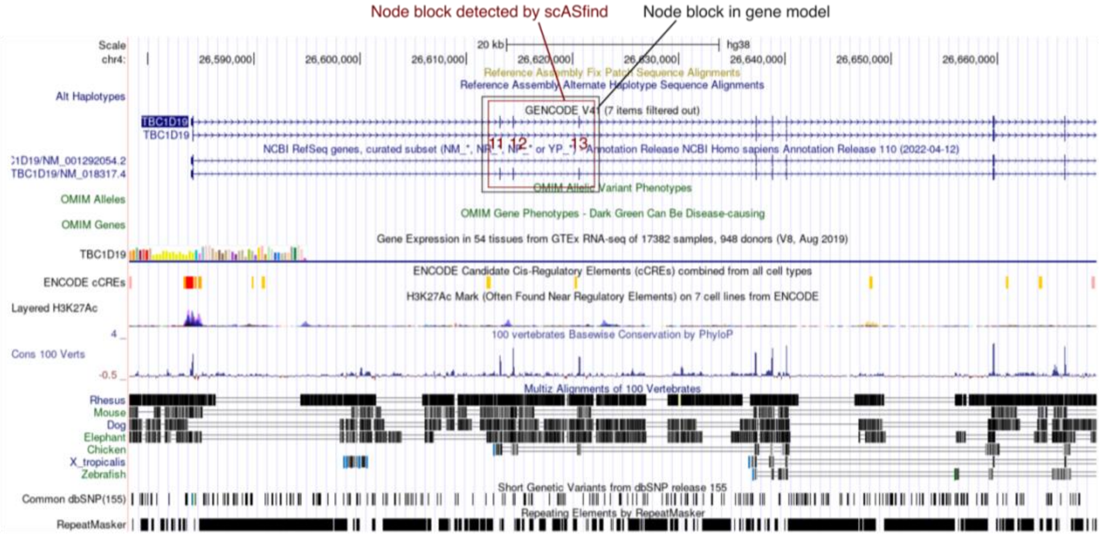

Fig S7: Reference assembly of *TBC1D19* in the human genome from the UCSC genome browser. Showing the two alternative isoforms with node blocks corresponding to scASfind results. Node block detected by scASfind is indicated in a dark red box while the node block in the gene annotation is shown with a black box.

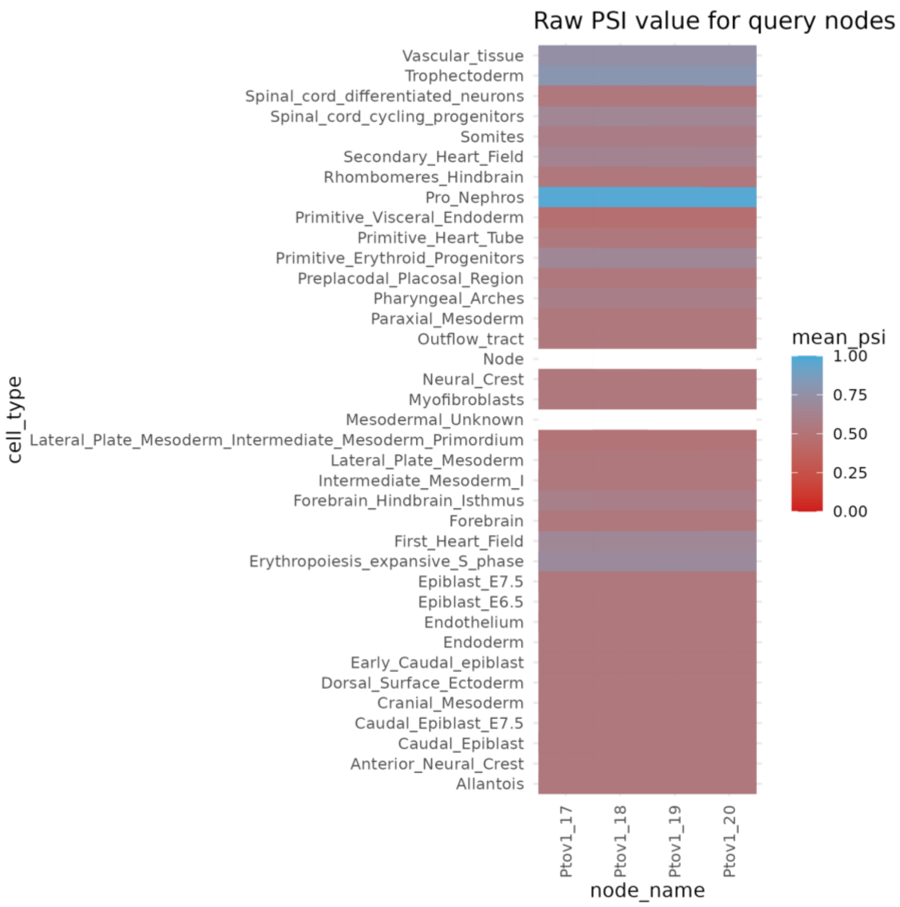

Fig S8: Raw PSI of node block in *Ptov1*. Showing inclusion of node *Ptov1_17-Ptov1_20* in Pro_Nephros in the mouse embryo data (6).

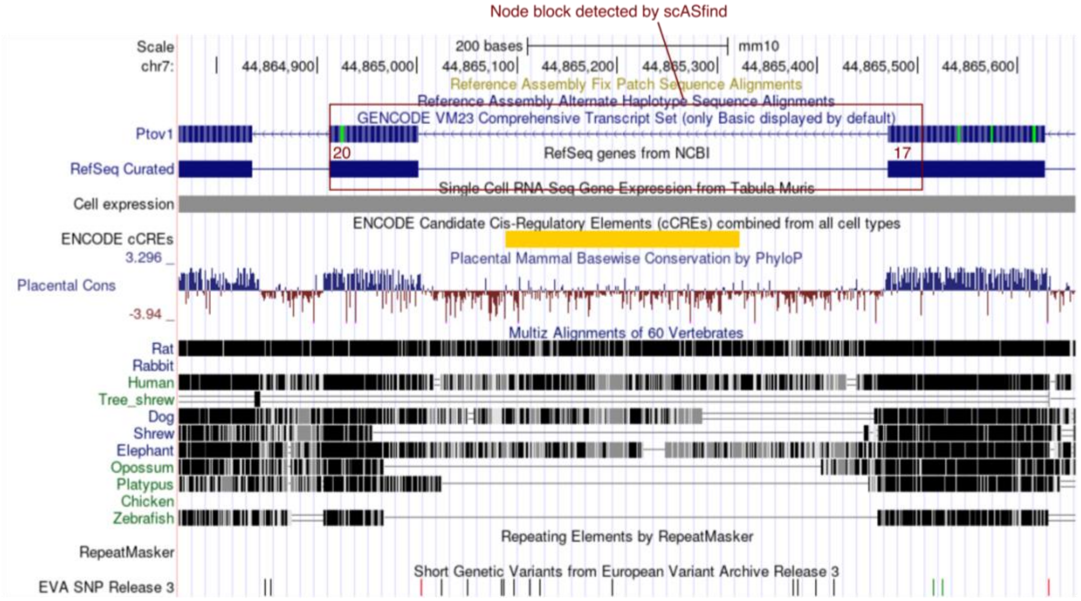

Fig S9: Ptov1 gene in mouse genome from the UCSC genome browser. Blue highlighted region is the detected node block by scASfind that has no documented isoform in

the mm10 reference genome. Node block detected by scASfind is indicated in a dark red box.

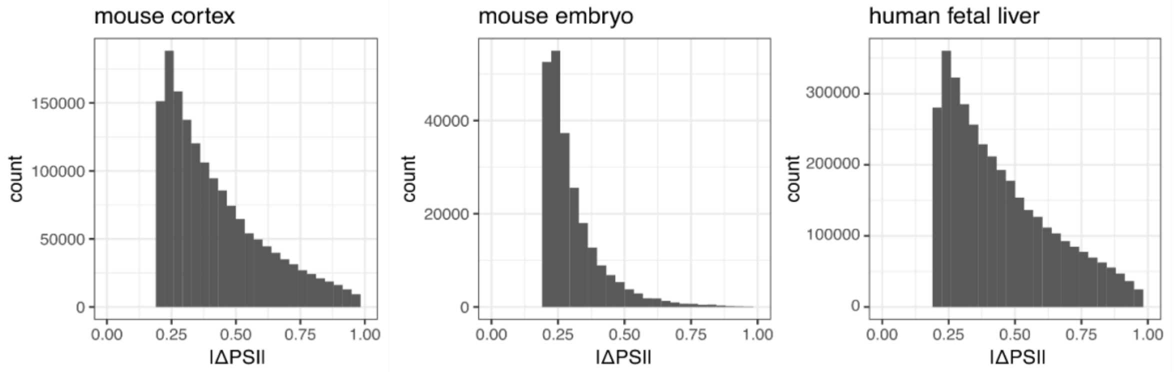

Fig S10: Histogram of PSI deviation from mean in the three datasets in all genes and all cell pools. Only showing encoded events which requires |ΔPSI| > 0.2. The histograms suggest that ΔPSI can be approximated by a lognormal distribution., PSI, percent spliced-in.

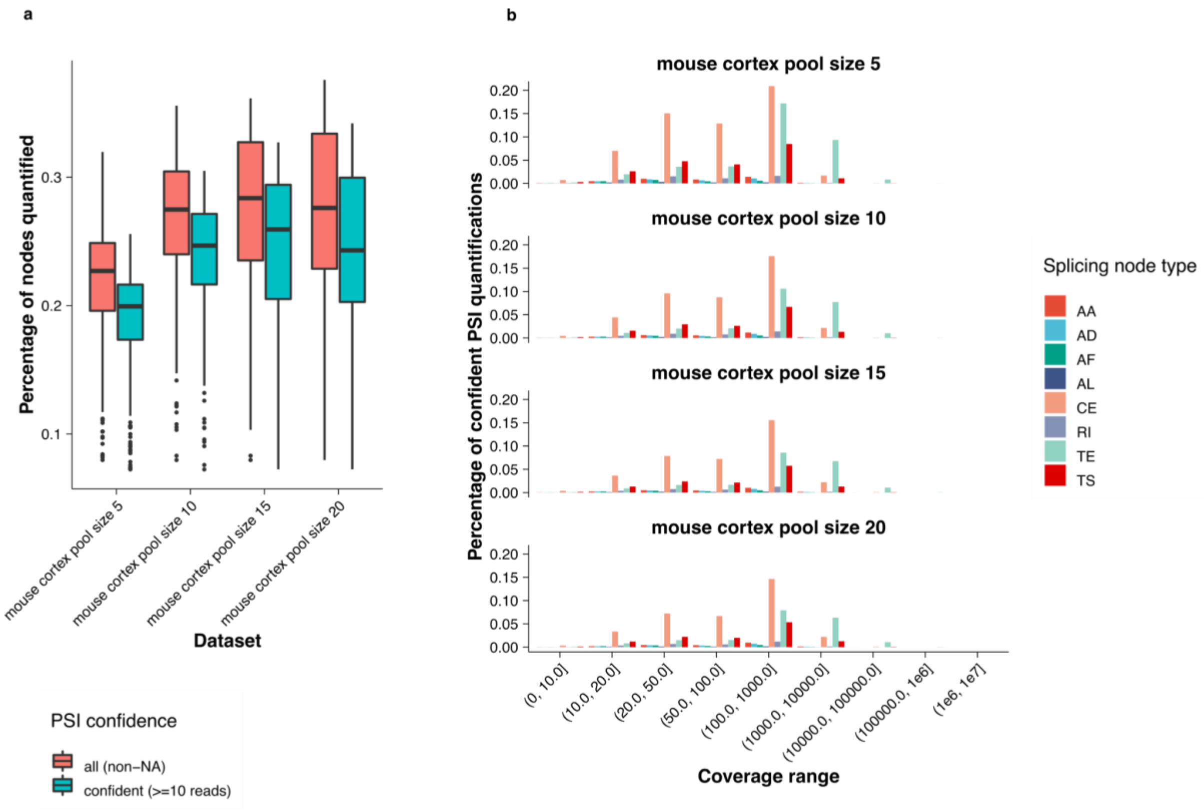

Fig S11 Impact of pool size on splicing quantification. Analysis is performed using the mouse cortex dataset. (a) Fraction of nodes with PSI quantification at different pool sizes. The bar in the boxplot shows the arithmetic mean, lower and upper hinges correspond to the first and third quartiles, whiskers extend from the hinge to the largest value no further than 1.5 * interquartile range and outliers beyond this range are plotted as individual data points.

(b) The read count distribution for splicing nodes which were confidently quantified in different pool sizes, respective to splicing node type. PSI, percent spliced-in.

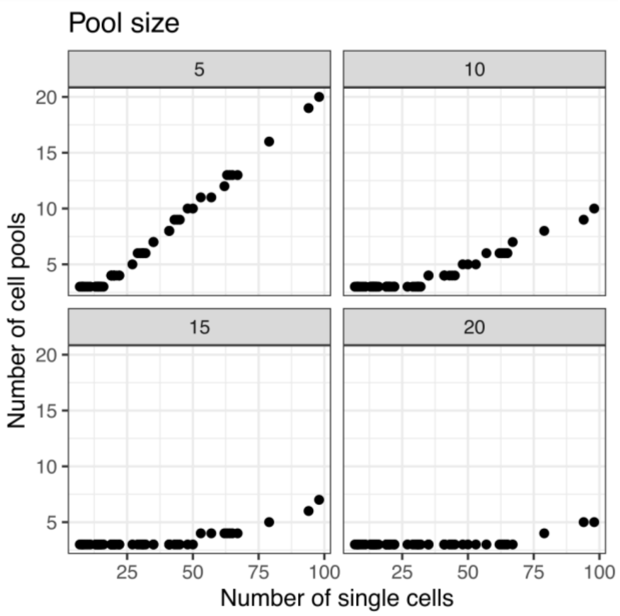

Fig S12 Comparison of the number of single cells and number of cell pools per cell type respective to different pool sizes. Note that the cell pooling approach requires a minimum 3 pools per cell type. Therefore, fewer cell types can achieve this limit as the pool size increases.

References

1. Wen WX, Mead AJ, Thongjuea S. MARVEL: an integrated alternative splicing analysis platform for single-cell RNA sequencing data. Nucleic Acids Res. 2023 Mar 21;51(5):e29.

2. Song Y, Parada GE, Lee JTH, Hemberg M. Mining alternative splicing patterns in scRNA-seq data using scASfind [Internet]. scASfind. 2023. Available from: https://github.com/hemberg-lab/scASfind. DOI 10.5281/zenodo.8241681.

3. Tasic B, Menon V, Nguyen TN, Kim TK, Jarsky T, Yao Z, et al. Adult mouse cortical cell taxonomy revealed by single cell transcriptomics. Nat Neurosci. 2016 Feb;19(2):335–46.

4. Tapial J, Ha KCH, Sterne-Weiler T, Gohr A, Braunschweig U, Hermoso-Pulido A, et al. An atlas of alternative splicing profiles and functional associations reveals new regulatory programs and genes that simultaneously express multiple major isoforms. Genome Res. 2017 Oct;27(10):1759–68.

5. Karolchik D, Hinrichs AS, Kent WJ. The UCSC Genome Browser. Curr Protoc Bioinformatics. 2009 Dec;Chapter 1:Unit1.4.

6. Salmen F, De Jonghe J, Kaminski TS, Alemany A, Parada GE, Verity-Legg J, et al. High-throughput total RNA sequencing in single cells using VASA-seq. Nat Biotechnol. 2022 Jun 27;1–14.
