# Supplementary material for: Mining alternative splicing patterns in scRNA-seq data using scASfind
Source: Genome Biol. 2024 Jul 29;25:197. doi: 10.1186/s13059-024-03323-6 (PMC11285346; doi:10.1186/s13059-024-03323-6)
Supplement: Supplementary file 2 — Additional file 2: Peer review history [file 13059_2024_3323_MOESM2_ESM.docx]

**Review history**

**First round of review**

**Reviewer 1**

The paper by Song et al reports a new tool to detect splicing patterns/ events which could represent candidate cell type markers. The paper employs a number of existing pipelines mostly by the same group to create an efficiently searchable data structure which compresses information over splicing events present in cell types in scRNA-seq experiments. Overall, the paper is well written and does a good job of detailing the software and its possible uses. Many possible questions could be asked at the methodological level, but as a software tool my impression is that it is easily accessible and well documented, so it could indeed represent a valid addition. My main comments for improvement are the following:
- Major: the authors state that the default size of the cell pool is 5, but then in the experiments they actually use three different sizes for three different data sets (5, 10, and 200, so varying over two orders of magnitude). Clearly this is a critical parameter which cannot be left as default and criteria as to why the authors set it to such widely differing values should be clearly articulated.
- Minor but worth discussing: the size of the cell pool will influence in a non-trivial way the variability in the error estimates of PSI. Because as far as I can tell the authors do not employ a tailored single-cell method for quantifying PSI (even though they do reference the existence of such methods), sufficient coverage of splice junctions will be needed and here the number of cells that get aggregated will play a major role. I would be grateful if the authors could expand on this topic as a way to emphasise that some awareness of design choices will certainly be required also of potential users.
- Minor but worth discussing: in general I am missing a discussion of the role of uncertainties. I already mentioned the sparsity/ cell pool size possible source of uncertainty, but obviously also the cell type assignments are generally not perfect, and this for rare cell types would potentially be an issue. This is particularly relevant since the authors emphasise that the high specificity of splicing markers make them suitable for rare cell type identification.

**Reviewer 2**

In their manuscript titled "Mining alternative splicing patterns in scRNA-seq data using scASfind," Song et al. present scASfind as a novel approach to identifying alternative splicing events from single-cell sequencing data. scASfind represents an advance in AS detection in single-cell analysis, in that it does not reply in imputation or model fitting. Several cells of the same type are combined into pools to provide sufficient read-depth to calculate PSI values with Whippet via MicroExonator and construct a splicing node x cell pool as well as a differential PSI matrix. Using the indexing approach from scfind, the PSI matrices are then compressed, enabling rapid access and complex searches. scASfind includes detection of MXE as well as blocks of multiple consecutive exons. The authors apply this tool to three diverse and independently-generated datasets. This is an important field, and the development of tools for detection of AS events in single-cell data are necessary as technology allows more accurate generation of long-read single-cell datasets. There is great potential in such data to advance understanding of cell-type specific transcriptomes and changes in development and disease. The tool presented in this manuscript is a useful contribution to the evolution of AS detection approaches, and will be of interest to the sc-tool development community as well as to biologists looking to analyze and detect AS events in their single cell data. However, the comparison of scASfind results to existing methods as well as the limitations of scASfind could be better addressed. I have the following suggestions to improve the manuscript:

The base analysis of scASfind depends upon Whippet. Although Whippet is a well-established tool in the AS field, it has several known limitations, including a reliance on annotated events, detection of fewer events, and higher FPR and FDR rates than other tools. These limitations are not presented or discussed in the current manuscript, but are important in the context of the number of high-confidence events the authors identify in their datasets and in considering the sensitivity of scASfind. The authors should address these considerations.

In the introduction, the authors provide a summary of existing tools to analyze AS events in single cell data, such as SingleSplice, Expedition, BRIE2, SpliZ and MARVEL. How does scASfind compare to at least some of these existing tools? A comparison of total number of events detected, FDR and FPR could be shown, for example, for analysis of the same dataset. These other methods may not be directly comparable due to differences in how events are detected, but it should be possible to analyze the same dataset and determine how many total events are called with similar thresholds, events in how many genes are detected and what the FDR rates are controlled at? In other words, how reliable are scASfind results compared to existing approaches?

An expanded presentation/discussion of the cell pooling approach is warranted, in particular to its application to AS analysis. In the main text, this is mentioned in simple, vague terms (for example page 4, lines 2-5 or in the methods page 18, line 52 to page 19 line 7). One has to search to find the number of cells pooled for each sample in the legend to extended data table 1. This information could be included in the methods. The column labeled "number of cell pools" in extended data table 1 is also duplicated. More generally, how is the number of pools determined, and how does this influence the results of scASfind? In the three datasets analyzed, there are ~7, 5.2 and 19.6 pools per cell type. Does this reflect the biological diversity of cells in the datasets or the technical parameters used to generate cell clusters and annotate cluster identity, and how does this difference in pool depth affect detection of AS events and initial PSI calculation in Whippet? For potential use of this package and biological interpretation of results, this is an important consideration.

What is the average read depth per cell and what is the read depth per event analyzed in the three datasets? The authors use a threshold of 10 counts per event for inclusion in the dataset, which is reasonable, but how many cell pools actually meet this criteria? Are most events in the range of 10-20 counts, 100 counts or more? How many genes were detected as expressed per cell in the datasets analyzed, that are even available for detection of AS events? Stated in another way, how high must a gene be expressed in a single cell to detect 10 reads per junction, and how deep must a cell be sequenced on average to obtain that level of coverage? From the biological perspective, the performance of the scASfind tool also must be considered with respect to the quality parameters of the starting single-cell dataset(s).

Page 7, lines 40-45. Can the authors add a paragraph to the discussion placing this conclusion in the context of the literature and existing single-cell analysis methods? The higher cell-type specificity for AS events rather than gene expression is an important finding and an important potential application for this tool. The discussion could be expanded to consider the MXE results and the consecutive exon splicing results from both the computational and biological perspectives. In addition, several lines in the discussion could use a citation, including Page 17 lines 39, 44 and 47 and Page 18 lines 7 and 12.

Page 13, line 57. This statement is inaccurate. The impact of a single splice event may be limited, however there are many examples where a single event can dramatically affect protein function, switching Mef2a from an activator to a repressor, for example, or changing the biophysical properties of the interaction between actin and myosin when the single event alters the myosin head structure. Splicing of both single events as well as coordinated, consecutive blocks of exons are important aspects of AS regulation.

The authors present several examples of node blocks they detect that are consistent with annotated isoforms. It would be helpful if the relevant literature where each of these isoforms and their cell-type specific function is cited. In addition, the extended data figures related to these events (Ext. Data Figures 1, 2, 3 & 4), should all be annotated either with exon numbers or to clearly highlight the node blocks and how they relate to the annotation. I can see there are different isoforms in the annotation, but it is not as clear to figure out how the annotation in the supplement maps onto the node blocks discussed in the main text.

Related to the detection of diverse isoform switching events, is there a comparable short-read transcriptome dataset quantifying AS events, to compare to the scASfind results to estimate how many or what percent of events detected in bulk RNA seq are detectable by scASfind in single-cell long-read seq data? The increased resolution of cell-type specific splice events may be a motivation to perform single-cell versus bulk seq, even if the overall number of events detected is lower. However, some estimate of this would be beneficial.

Figure 6 - Please adjust the alpha values on the bar plots, as currently they are difficult to see (panels A-C). The bars are so light and faint, it is difficult to see the differences in shading as well as the length of the bars. This is also true of the light grey to dark grey shading of the splice junctions in the Sashimi plots and the read counts in Figure 7 panels A and B. It is hard to see the splice junctions. Also for Figure 7, please provide a description for what the different shades of grey represent (the sample?)? It is not clear what event the PSI value refers to in Figure 7 - is this the average for all junctions in the splicing node? What it the standard deviation of this mean PSI?

**Authors’ response to reviewers**

**Reviewer #1: The paper by Song et al reports a new tool to detect splicing patterns/ events which could represent candidate cell type markers. The paper employs a number of existing pipelines mostly by the same group to create an efficiently searchable data structure which compresses information over splicing events present in cell types in scRNA-seq experiments. Overall, the paper is well written and does a good job of detailing the software and its possible uses. Many possible questions could be asked at the methodological level, but as a software tool my impression is that it is easily accessible and well documented, so it could indeed represent a valid addition. My main comments for improvement are the following:**

Thank you for the positive assessment of our manuscript and the suggestions that improved our manuscript.

1. **Major: the authors state that the default size of the cell pool is 5, but then in the experiments they actually use three different sizes for three different data sets (5, 10, and 200, so varying over two orders of magnitude). Clearly this is a critical parameter which cannot be left as default and criteria as to why the authors set it to such widely differing values should be clearly articulated.**

We appreciate the reviewer bringing up the importance of pool size. The pool size is an empirical parameter that is determined by the sequencing depth and the tissue. Choosing a pool size needs to balance two aspects: ensuring a proper coverage for PSI quantification, while preserving a sufficient number of cell pools per cell type to allow for a rigorous hypergeometric test for MXE and Node block detection.

We employed a much larger pool size (200) in the mouse embryo data because it is much sparser than the mouse brain data. In the mouse embryo data, the total count per single cell was 14,936±12,319 (mean±std), while it was 1,594,655±491,715 in the mouse brain data (pool size 5 used), and 451,901±393,948 in the human fetal liver data (pool size 10 used). There were also more single cells per cell type in the mouse embryo data to allow for a large pool size while having sufficient pools per cell type.

We added the sequencing depth and pool size information used in this study in Table 1. We also added the below paragraph in the first section of the results to explain how to determine pool size:

“The size of cell pools is an important hyperparameter in the analysis, which should be carefully determined on a per-case basis. Pooling single cells aims to reduce the technical sparsity of scRNA-seq data, which could be due to technology or tissue. In our examples, the dataset by VASA-seq (mouse embryo) was two magnitudes sparser than the two Smart-seq2 datasets (mouse cortex, human fetal liver), therefore we employed a much larger pool size (Table 1). Choosing a pool size needs to balance two aspects: ensuring a proper coverage for PSI quantification, while preserving enough cell pools per cell type to faithfully represent the variation of cell abundance and allow effective statistics tests in scASfind. An examination of the impact of pool size on scASfind results in the mouse cortex data can be found in the methods section.“

2. **Minor but worth discussing: the size of the cell pool will influence in a non-trivial way the variability in the error estimates of PSI. Because as far as I can tell the authors do not employ a tailored single-cell method for quantifying PSI (even though they do reference the existence of such methods), sufficient coverage of splice junctions will be needed and here the number of cells that get aggregated will play a major role. I would be grateful if the authors could expand on this topic as a way to emphasise that some awareness of design choices will certainly be required also of potential users.**

We thank the reviewer for pointing out the impact of cell pool size on the error estimation of PSI. Indeed, we employ Whippet for PSI quantification, and reduce the data sparsity of scRNA-seq by making cell pools. The sequencing depth will influence the PSI quantification efficiency, but having more cells per pool means fewer cell pools per cell type, which can negatively impact the statistical tests used in scASfind. Therefore, it is worth investigating whether a larger cell pool always lead to more confidently quantified splicing nodes, and how cell pool size impact scASfind results via number of pools per cell type.

To examine the impact of pool size on scASfind results, we ran the scASfind analysis using a range of pool sizes (5, 10, 15, 20) on the mouse cortex dataset. We compared the PSI quantification coverage, the number of pools per cell type as well as the scASfind results including marker nodes, MXE events and node blocks. We summarized the following analysis and added it into the manuscript.

First, we examined the percentage of nodes quantified among all annotated nodes (Figure R1a). We found that despite a slight increase in the total percentage of nodes quantified as the pool size increased, the percentage of confidently quantified nodes (with >=10 reads) remained stable and saw a slight decrease at pool size 20. This is further supported by the fact that the coverage distribution of confidently quantified nodes remained similar across the pool sizes (Figure R1b). Additionally, comparing the percentage of nodes quantified among all nodes in the three dataset suggested that the selected pool sizes gave comparable numbers of confidently quantified nodes: 20.47±4.30; 30.63±13.78; 15.72±5.63 (mean±stddev) .

We then observed the relationship between the number of single cells, and the number of cell pools per cell type (Figure R2). It is clear that at pool size 5, the number of cell pools has an almost linear relationship with the number of single cells, which adequately reflects the biological variation. With increased pool size, more and more cell types would have 3 cell pools, which is the minimum number of cell pools required by the tool. Therefore, from the perspective of maintaining the single cell type proportion in the dataset, using pool size 5 is appropriate. This is also important for the hypergeometric tests employed both in MXE and node block detection, where a sufficient number of pools per cell type is required to make the test effective.

In summary, using a pool size 5 for the mouse cortex data ensures a balance between achieving sufficient coverage for PSI quantification, and retaining the abundance variation between cell types.

**Figure R1: Impact of pool size on splicing quantification.** Analysis is performed using the mouse cortex dataset. (a) Fraction of nodes with PSI quantification at different pool sizes. The bar in the boxplot shows the arithmetic mean, lower and upper hinges correspond to the first and third quartiles, whiskers extend from the hinge to the largest value no further than 1.5 * interquartile range and outliers beyond this range are plotted as individual data points. (b) The read count distribution for splicing nodes which were confidently quantified in different pool sizes, respective to splicing node type.

**Figure R2: Comparison of the number of single cells and number of cell pools per cell type respective to different pool sizes.** Note that the cell pooling approach requires a minimum 3 pools per cell type. Therefore, fewer cell types can achieve this limit as the pool size increases.

3. **Minor but worth discussing: in general I am missing a discussion of the role of uncertainties. I already mentioned the sparsity/ cell pool size possible source of uncertainty, but obviously also the cell type assignments are generally not perfect, and this for rare cell types would potentially be an issue. This is particularly relevant since the authors emphasise that the high specificity of splicing markers make them suitable for rare cell type identification.**

Thank you for bringing the uncertainties in cell type assignment to our attention. We agree that the cell type assignment errors may result in rare cell types that are annotated incorrectly. In the default scASfind analysis, the index object is created based on user-provided cell type annotations. The data indexing per cell type allows us to perform splicing pattern search across cell types and determine cell type specificity. Although it is possible to return the exact cell pools in which the query splicing pattern is presented for different cell types using the *findCellTypes* function, there is no guarantee that the cells which get pooled are transcriptomically uniform as the default pooling is random.

After rethinking this problem, we reckon that the high precision of splicing markers benefits rare cell type tracing, rather than rare cell type detection. Another conceptual challenge is whether rare cell types can be defined at splicing level. Currently, methods for rare cell type detection typically aim to capture traces of meaningful differences on gene expression (Wang et al., 2024; Wegmann et al., 2019). This provides a further opportunity to use the rare cell types detected by such algorithms and find splicing patterns specific to these cell types using scASfind, which can achieve higher precision and might be a better marker to validate these cell types experimentally.

On balance, we addressed these challenges in the discussion section of the revised manuscript:

“Current single-cell platforms typically focus on sequencing only the 3’ or 5’ ends of transcripts, leaving alternative splicing largely unexplored at the single-cell level. Our analysis of the scASfind splicing node markers strongly suggests that single-node splicing patterns can provide higher cell type precision than gene expression. This is particularly relevant to tracing rare cell types. By leveraging rare cell types identified through specialized algorithms (S. Wang et al., 2024; Wegmann et al., 2019), we can explore specific splicing patterns using scASfind, offering potential for higher precision and experimental validation. For instance, studies have demonstrated subtype-specific splicing in neurons (Ling et al., 2020; Murphy et al., 2016; Sato et al., 2022), reinforcing the utility of splicing patterns in distinguishing neuron subtypes. Understanding AS events with strong cell population specificity is crucial for effectively studying cellular heterogeneity. Additionally, the systematic identification of MXEs and tissue-specific coordinated splicing events provides insights into cell type-specific AS regulation, enriching our understanding of the regulatory landscape. “

**Reviewer #2: In their manuscript titled "Mining alternative splicing patterns in scRNA-seq data using scASfind," Song et al. present scASfind as a novel approach to identifying alternative splicing events from single-cell sequencing data. scASfind represents an advance in AS detection in single-cell analysis, in that it does not reply in imputation or model fitting. Several cells of the same type are combined into pools to provide sufficient read-depth to calculate PSI values with Whippet via MicroExonator and construct a splicing node x cell pool as well as a differential PSI matrix. Using the indexing approach from scfind, the PSI matrices are then compressed, enabling rapid access and complex searches. scASfind includes detection of MXE as well as blocks of multiple consecutive exons. The authors apply this tool to three diverse and independently-generated datasets. This is an important field, and the development of tools for detection of AS events in single-cell data are necessary as technology allows more accurate generation of long-read single-cell datasets. There is great potential in such data to advance understanding of cell-type specific transcriptomes and changes in development and disease. The tool presented in this manuscript is a useful contribution to the evolution of AS detection approaches, and will be of interest to the sc-tool development community as well as to biologists looking to analyze and detect AS events in their single cell data. However, the comparison of scASfind results to existing methods as well as the limitations of scASfind could be better addressed. I have the following suggestions to improve the manuscript:**

We thank the reviewer for their positive view of our manuscript and detailed suggestions that significantly improved the manuscript.

1. **The base analysis of scASfind depends upon Whippet. Although Whippet is a well-established tool in the AS field, it has several known limitations, including a reliance on annotated events, detection of fewer events, and higher FPR and FDR rates than other tools. These limitations are not presented or discussed in the current manuscript, but are important in the context of the number of high-confidence events the authors identify in their datasets and in considering the sensitivity of scASfind. The authors should address these considerations.**

We appreciate the reviewer for pointing out that a discussion about the known limitations of Whippet and we agree that it is highly relevant to the scASfind analysis. We summarized the limitations mentioned by Whippet authors and a benchmark study, and added the following paragraph to the manuscript to address these considerations:

“The PSI quantification used by scASfind comes from the event-level splicing quantification tool Whippet. Whippet is an established method, and it has been shown to be efficient with high recall in a benchmark study (Fenn et al., 2023), but it has several limitations. The algorithm only detects and analyzes annotated AS events in its contiguous splice graphs index (Sterne-Weiler et al., 2018), and it is less effective on detecting events *de novo* (Fenn et al., 2023). In our study scASfind only detected splicing patterns of nodes in the Whippet index.”

2. **In the introduction, the authors provide a summary of existing tools to analyze AS events in single cell data, such as SingleSplice, Expedition, BRIE2, SpliZ and MARVEL. How does scASfind compare to at least some of these existing tools? A comparison of total number of events detected, FDR and FPR could be shown, for example, for analysis of the same dataset. These other methods may not be directly comparable due to differences in how events are detected, but it should be possible to analyze the same dataset and determine how many total events are called with similar thresholds, events in how many genes are detected and what the FDR rates are controlled at? In other words, how reliable are scASfind results compared to existing approaches?**

We thank the reviewer's suggestion on comparing scASfind results with some of the existing tools for AS analysis in single cell data. We compared scASfind results with MARVEL (Wen et al., 2023) using the mouse cortex dataset to better understand how our method differs. We summarized the following analysis and added it into the manuscript.

We first compared the cell type-specific splicing events detected by MARVEL with the cell type marker nodes from scASfind. The principles behind the two methods are fairly different. MARVEL differential splicing is designed for pairwise comparisons and uses Anderson-Darling or the D Test Statistics for comparing the overall PSI distribution. On the other hand, scASfind returns a user-given number of top marker nodes ranked by F1 score. To ensure a fair comparison, we adopted the following strategy.

We carried out pairwise comparisons of all 1,176 pairs of cell types in the mouse cortex data. We then applied the CompareValues function from MARVEL to obtain differential splicing events between these cell type pairs. We also applied the cellTypeMarker function from scASfind to compute the above and below markers of the same cell type pairs, using only the two cell types being compared as the background to achieve pairwise comparison. We filtered for significantly differentially spliced skipped exon (SE) events in MARVEL, then assigned those that have an ‘Included’ modality in one of the cell types and an ‘Excluded’ modality in the other to be the positive marker of the first cell type, and vice versa. scASfind markers were filtered to include only core exon (CE) type and ranked by F1 score. Finally, we observed the ranking and F1 score of these marker SEs from MARVEL in scASfind.

We observed a remarkable degree of consistency between the two tools. Among the 1,176 pairs of cell types, MARVEL returned a total of 441 SE markers for 318 cell type pairs. Among the MARVEL SE markers, 232 were ranked top 1 by scASfind and 330 were among the top 5 (Figure R3). For those marker nodes that were less significant according to MARVEL, we also found lower ranking and F1 score in scASfind (Figure R3). The comparison suggested that MARVEL focused on returning the strongest signal while scASfind provided more information, reporting events in a custom range of F1. Both tools are highly consistent in capturing the strongest signal from the splicing nodes that maximally distinguish a pair of cell types. Nevertheless, scASfind can provide results for all cell type pairs while many of these pairs did not have significant differentially spliced SEs in MARVEL analysis.

**Figure R3 Comparison of MARVEL differentially spliced SEs with scASfind CE markers.** Each dot represents a core exon detected by both methods as differentially spliced between a pair of cell types in the mouse cortex data. The dot size corresponds to the F1 score in scASfind, and the count of dots per scASfind rank is shown in the bar chart.

Using a similar strategy, we further compared the cell type specific MXE events found by MARVEL and scASfind. MARVEL found 9 pairs of MXE, among which 5 pairs were detected by scASfind (Gria1, chr11:57309769:57309883-chr11:57310608:57310722; Gria2, chr3:80691320:80691434-chr3:80690404:80690518; Gria3, chrX:41654810-41654924-chrX:41669501:41669615; Acsl6, chr11:54336936:54337013-chr11:54337071:54337148; Snap25, chr2:136769743:136769860-chr2:136770057:136770174), 3 pairs that one of the nodes were not quantified in scASfind, and one pair in which there were no cell types showing significance. On the other hand, scASfind found 63 pairs of MXEs in total and 14 highly confident ones. It is worth noting that the scASfind MXEs were calculated in a one-to-all way while MARVEL calculations were pairwise. Therefore, the consensus of MXE events between scASfind and MARVEL is still strong.

3. **An expanded presentation/discussion of the cell pooling approach is warranted, in particular to its application to AS analysis. In the main text, this is mentioned in simple, vague terms (for example page 4, lines 2-5 or in the methods page 18, line 52 to page 19 line 7). One has to search to find the number of cells pooled for each sample in the legend to extended data table 1. This information could be included in the methods. The column labeled "number of cell pools" in extended data table 1 is also duplicated. More generally, how is the number of pools determined, and how does this influence the results of scASfind? In the three datasets analyzed, there are ~7, 5.2 and 19.6 pools per cell type. Does this reflect the biological diversity of cells in the datasets or the technical parameters used to generate cell clusters and annotate cluster identity, and how does this difference in pool depth affect detection of AS events and initial PSI calculation in Whippet? For potential use of this package and biological interpretation of results, this is an important consideration.**

We appreciate the reviewer highlighting the importance of clarifying the cell pooling approach. We have added a detailed explanation of how to choose the cell pool size, performed further analysis, and modified the manuscript accordingly.

The sequencing depth will influence the PSI quantification efficiency, but having more cells per pool means fewer cell pools per cell type, which can negatively impact the statistical tests used in scASfind. Therefore, it is worth investigating whether a larger cell pool always lead to more confidently quantified splicing nodes, and how cell pool size impact scASfind results via number of pools per cell type.

To examine the impact of pool size on scASfind results, we ran the scASfind analysis using a range of pool sizes (5, 10, 15, 20) on the mouse cortex dataset. We compared the PSI quantification coverage, the number of pools per cell type as well as the scASfind results including marker nodes, MXE events and node blocks. We summarized the following analysis and added it into the manuscript.

First, we examined the percentage of nodes quantified among all annotated nodes (Figure R4a). We found that despite a slight increase in the total percentage of nodes quantified as the pool size increased, the percentage of confidently quantified nodes (with >=10 reads) remained stable and saw a slight decrease at pool size 20. This is further supported by the fact that the coverage distribution of confidently quantified nodes remained similar across the pool sizes (Figure R4b). Additionally, comparing the percentage of nodes quantified among all nodes in the three dataset suggested that the selected pool sizes gave comparable numbers of confidently quantified nodes: 20.47±4.30; 30.63±13.78; 15.72±5.63 (mean±stddev).

We then observed the relationship between the number of single cells, and the number of cell pools per cell type (Figure R5). It is clear that at pool size 5, the number of cell pools has an almost linear relationship with the number of single cells, which adequately reflects the biological variation. With increased pool size, more and more cell types would have 3 cell pools, which is the minimum number of cell pools required by the tool. Therefore, from the perspective of maintaining the single cell type proportion in the dataset, using pool size 5 is appropriate. This is also important for the hypergeometric tests employed both in MXE and node block detection where a sufficient number of pools per cell type is required to make the test effective.

In summary, using a pool size 5 for the mouse cortex data ensures a balance between achieving sufficient coverage for PSI quantification, and retaining the abundance variation between cell types.

**Figure R4: Impact of pool size on splicing quantification.** Analysis is performed using the mouse cortex dataset. (a) Fraction of nodes with PSI quantification at different pool sizes. The bar in the boxplot shows the arithmetic mean, lower and upper hinges correspond to the first and third quartiles, whiskers extend from the hinge to the largest value no further than 1.5 * interquartile range and outliers beyond this range are plotted as individual data points. (b) The read count distribution for splicing nodes which were confidently quantified in different pool sizes, respective to splicing node type.

**Figure R5: Comparison of the number of single cells and number of cell pools per cell type respective to different pool sizes.** Note that the cell pooling approach requires a minimum 3 pools per cell type. Therefore, fewer cell types can achieve this limit as the pool size increases.

4. **What is the average read depth per cell and what is the read depth per event analyzed in the three datasets? The authors use a threshold of 10 counts per event for inclusion in the dataset, which is reasonable, but how many cell pools actually meet this criteria? Are most events in the range of 10-20 counts, 100 counts or more? How many genes were detected as expressed per cell in the datasets analyzed, that are even available for detection of AS events? Stated in another way, how high must a gene be expressed in a single cell to detect 10 reads per junction, and how deep must a cell be sequenced on average to obtain that level of coverage? From the biological perspective, the performance of the scASfind tool also must be considered with respect to the quality parameters of the starting single-cell dataset(s).**

Thank you for bringing forward several important points regarding read depth and expression value. We performed further analysis to address these questions:

We showed in Figure R4b that most junctions achieved a read count between 100-1,000 for cell pool size 5 in the mouse cortex data, with variations among splicing node types. We further analyzed the gene expression level required for having different percentages of splicing nodes being confidently quantified (Figure R6) for cell pool size 5 in the mouse cortex data. We observed that highly expressed genes tend to have a higher percentage of confidently quantified nodes. Nevertheless, the distribution of the number of genes that achieved different percentages of confidently quantified nodes mostly overlapped.

**Figure R6: Fraction of nodes with confident PSI quantification in gene.** Showing the distribution of gene expression level (TpM) for genes with different numbers of splicing nodes, respective to different fractions of confidently quantified nodes. Splicing odes that had no less than 10 read counts were considered confidently quantified. TpM, transcript per million.

5. **Page 7, lines 40-45. Can the authors add a paragraph to the discussion placing this conclusion in the context of the literature and existing single-cell analysis methods? The higher cell-type specificity for AS events rather than gene expression is an important finding and an important potential application for this tool. The discussion could be expanded to consider the MXE results and the consecutive exon splicing results from both the computational and biological perspectives. In addition, several lines in the discussion could use a citation, including Page 17 lines 39, 44 and 47 and Page 18 lines 7 and 12.**

We thank the reviewer for the suggestion on the high cell type specificity of splicing events. We added the following paragraph in discussions:

“Current single-cell platforms typically focus on sequencing only the 3’ or 5’ ends of transcripts, leaving alternative splicing largely unexplored at the single-cell level. Our analysis of the scASfind splicing node markers strongly suggests that single-node splicing patterns can provide higher cell type precision than gene expression. This is particularly relevant to tracing rare cell types. By leveraging rare cell types identified through specialized algorithms (S. Wang et al., 2024; Wegmann et al., 2019), we can explore specific splicing patterns using scASfind, offering potential for higher precision and experimental validation. For instance, studies have demonstrated subtype-specific splicing in neurons (Ling et al., 2020; Murphy et al., 2016; Sato et al., 2022), reinforcing the utility of splicing patterns in distinguishing neuron subtypes. Understanding AS events with strong cell population specificity is crucial for effectively studying cellular heterogeneity. Additionally, the systematic identification of MXEs and tissue-specific coordinated splicing events provides insights into cell type-specific AS regulation, enriching our understanding of the regulatory landscape. “

Relevant references have been included for the mentioned lines, and these discussions were also re-written to improve clarity.

6. **Page 13, line 57. This statement is inaccurate. The impact of a single splice event may be limited, however there are many examples where a single event can dramatically affect protein function, switching Mef2a from an activator to a repressor, for example, or changing the biophysical properties of the interaction between actin and myosin when the single event alters the myosin head structure. Splicing of both single events as well as coordinated, consecutive blocks of exons are important aspects of AS regulation.**

We thank the reviewer for the feedback and have amended the manuscript accordingly:

“By definition, the splicing nodes considered in scASfind only involve a single event. Though a single event can lead to drastic shifts of gene function (M. Zhang et al., 2015), splicing events are often coordinated, resulting in multiple consecutive exons being simultaneously included or excluded.”

7. **The authors present several examples of node blocks they detect that are consistent with annotated isoforms. It would be helpful if the relevant literature where each of these isoforms and their cell-type specific function is cited. In addition, the extended data figures related to these events (Ext. Data Figures 1, 2, 3 & 4), should all be annotated either with exon numbers or to clearly highlight the node blocks and how they relate to the annotation. I can see there are different isoforms in the annotation, but it is not as clear to figure out how the annotation in the supplement maps onto the node blocks discussed in the main text.**

We thank the reviewer for suggesting a clearer annotation of the node blocks in extended data figures. We have added the node number, the node block detected by scASfind, and the observed node block in the gene model onto Extended data Figure 1-4, 6.

8. **Related to the detection of diverse isoform switching events, is there a comparable short-read transcriptome dataset quantifying AS events, to compare to the scASfind results to estimate how many or what percent of events detected in bulk RNA seq are detectable by scASfind in single-cell long-read seq data? The increased resolution of cell-type specific splice events may be a motivation to perform single-cell versus bulk seq, even if the overall number of events detected is lower. However, some estimate of this would be beneficial.**

Thank you for suggesting this analysis. We utilized the PSI quantification in the VastDB (Tapial et al., 2017), an atlas of alternative splicing profiles in vertebrate cell and tissue types based on bulk RNA-seq data to compare with scASfind. Specifically, we analyzed the VastDB PSI values of scASfind marker splicing nodes detected in the mouse cortex data. We summarized the following analysis and added it into the manuscript.

We first focused on several glial cell types, as VastDB provided PSI quantification from bulk RNA-seq using purified cell type samples. From the mouse cortex data we identified the top 10 scASfind marker nodes (ranked by F1 score) among glial cell types. We then plotted the PSI values of these marker nodes from matching bulk cell type samples in VastDB and several additional brain tissues (Figure R7). It was clear that for microglia, oligodendrocytes and OPCs, the top scASfind marker nodes exhibited increased PSI in the respective VastDB cell type sample. Furthermore, we observed that cortical, cerebellar, and whole brain data, which encompass mixed neuronal and glial cell types, did not exhibit high PSI for these marker nodes. Therefore, scASfind results were well supported by the PSI quantification from purified cell types in VastDB. The analysis further highlights the enhanced resolution provided by single-cell results compared to bulk RNA-seq from mixed cell types.

**Figure R7: Evaluation of marker nodes from five cell types in the mouse brain data using VastDB.** For each tissue in VastDB we calculated the PSI for the top 10 cell type marker nodes reported by scASfind. The box encompasses the 25th-75th percentile and the whiskers extend to the 5th and 95th percentiles with outliers shown as dots. The box which should provide the best match based on the annotated labels is highlighted in green.

We then analyzed the VastDB PSI values of all single cell splicing markers in the mouse cortex data across all tissues. Specifically, we aim to determine whether these neuron or glial cell marker splicing nodes show neural patterns in bulk RNA-seq tissue data. We generated a matrix of VastDB PSI values using scASfind marker nodes as features, and VastDB samples as observations. By applying probabilistic PCA (for compatibility of NA values in VastDB PSI quantification) onto the scaled and centered matrix, we observed the different tissue’s position on the first 2 PCs (Figure R8). We found that the neuronal tissues were separated by PC2 using the scASfind marker node features, suggesting that the scASfind marker nodes indeed show neural specificity.

**Figure R8: scASfind markers in VastDB show neural specificity.** We calculated the top 500 marker splicing nodes for each cell type from scASfind analysis of the mouse cortex data. The VastDB PSI values of these marker nodes for various tissues were then subtracted and subjected to probabilistic principal component analysis. The first two principal components are represented, showing that neuronal tissues and immune cells are distinct from other tissue types. PC, principal component.

9. **Figure 6 - Please adjust the alpha values on the bar plots, as currently they are difficult to see (panels A-C). The bars are so light and faint, it is difficult to see the differences in shading as well as the length of the bars. This is also true of the light grey to dark grey shading of the splice junctions in the Sashimi plots and the read counts in Figure 7 panels A and B. It is hard to see the splice junctions. Also for Figure 7, please provide a description for what the different shades of grey represent (the sample?)? It is not clear what event the PSI value refers to in Figure 7 - is this the average for all junctions in the splicing node? What is the standard deviation of this mean PSI?**

Thank you for your suggestions on improving the clarity of visualizations. We adjusted the Figure 6 bar plot by increasing the maximum alpha value and adding a border. The different shades in Figure 7 were indeed meant to differentiate tissues and we added explanations to the caption. The PSI value indeed means the average PSI across all coordinated splice nodes. The respective standard deviations have been added to the figure.

**References**

Tapial, J., Ha, K. C. H., Sterne-Weiler, T., Gohr, A., Braunschweig, U., Hermoso-Pulido, A., Quesnel-Vallières, M., Permanyer, J., Sodaei, R., Marquez, Y., Cozzuto, L., Wang, X., Gómez-Velázquez, M., Rayon, T., Manzanares, M., Ponomarenko, J., Blencowe, B. J., & Irimia, M. (2017). An atlas of alternative splicing profiles and functional associations reveals new regulatory programs and genes that simultaneously express multiple major isoforms. *Genome Research*, *27*(10), 1759–1768.

Wang, S., Li, H., Zhang, K., Wu, H., Pang, S., Wu, W., Ye, L., Su, J., & Zhang, Y. (2024). scSID: A lightweight algorithm for identifying rare cell types by capturing differential expression from single-cell sequencing data. *Computational and Structural Biotechnology Journal*, *23*, 589–600.

Wegmann, R., Neri, M., Schuierer, S., Bilican, B., Hartkopf, H., Nigsch, F., Mapa, F., Waldt, A., Cuttat, R., Salick, M. R., Raymond, J., Kaykas, A., Roma, G., & Keller, C. G. (2019). CellSIUS provides sensitive and specific detection of rare cell populations from complex single-cell RNA-seq data. *Genome Biology*, *20*(1), 142.

Wen, W. X., Mead, A. J., & Thongjuea, S. (2023). MARVEL: an integrated alternative splicing analysis platform for single-cell RNA sequencing data. *Nucleic Acids Research*, *51*(5), e29.

**Second round of review**

**Reviewer 1**

I thank the authors for their thoughtful and exhaustive response

**Reviewer 2**

The authors have addressed my questions in their response and in the revised manuscript. The additional supplementary figures, analysis, and textual modifications have clarified multiple points and improved the manuscript. scASfind performs comparably to other approaches, and based on the comparison with MARVEL and vastDB, may offer advantages, thus fulfilling a need in the field. In addition, the authors have addressed several limitations of their approach improving the interpretability and accessibility of scASfind results.
